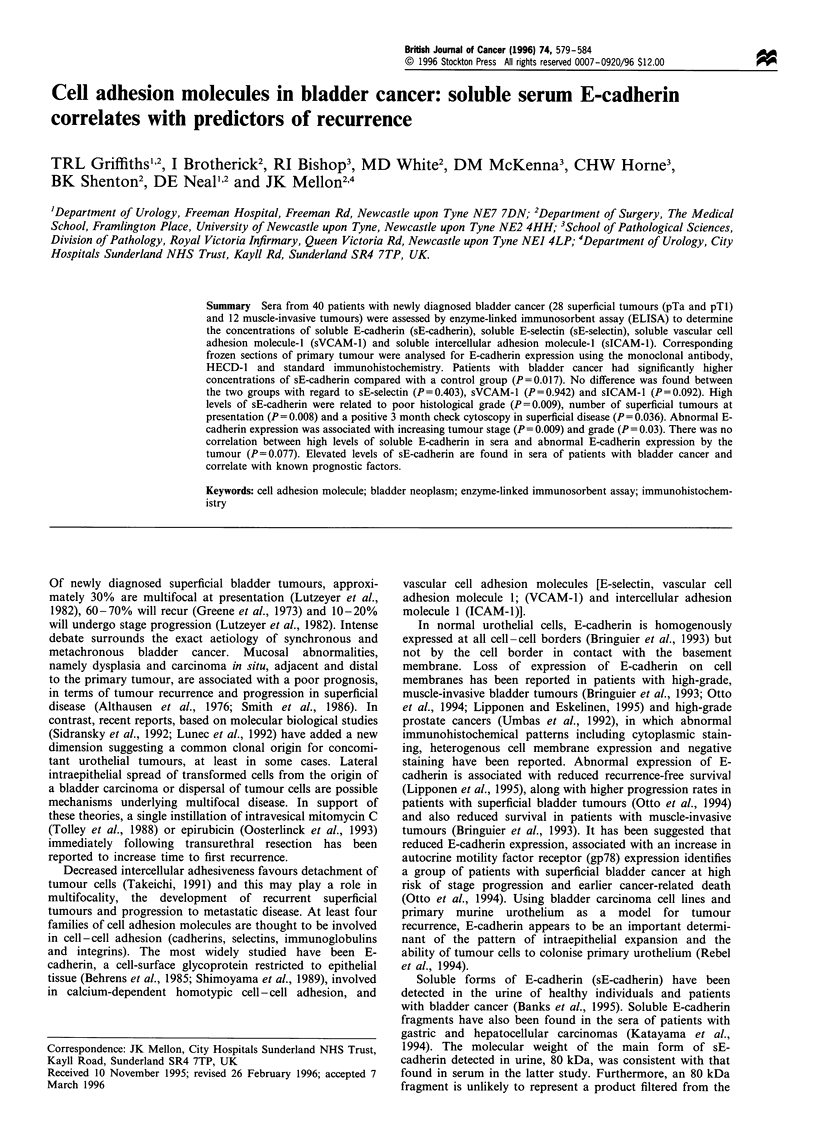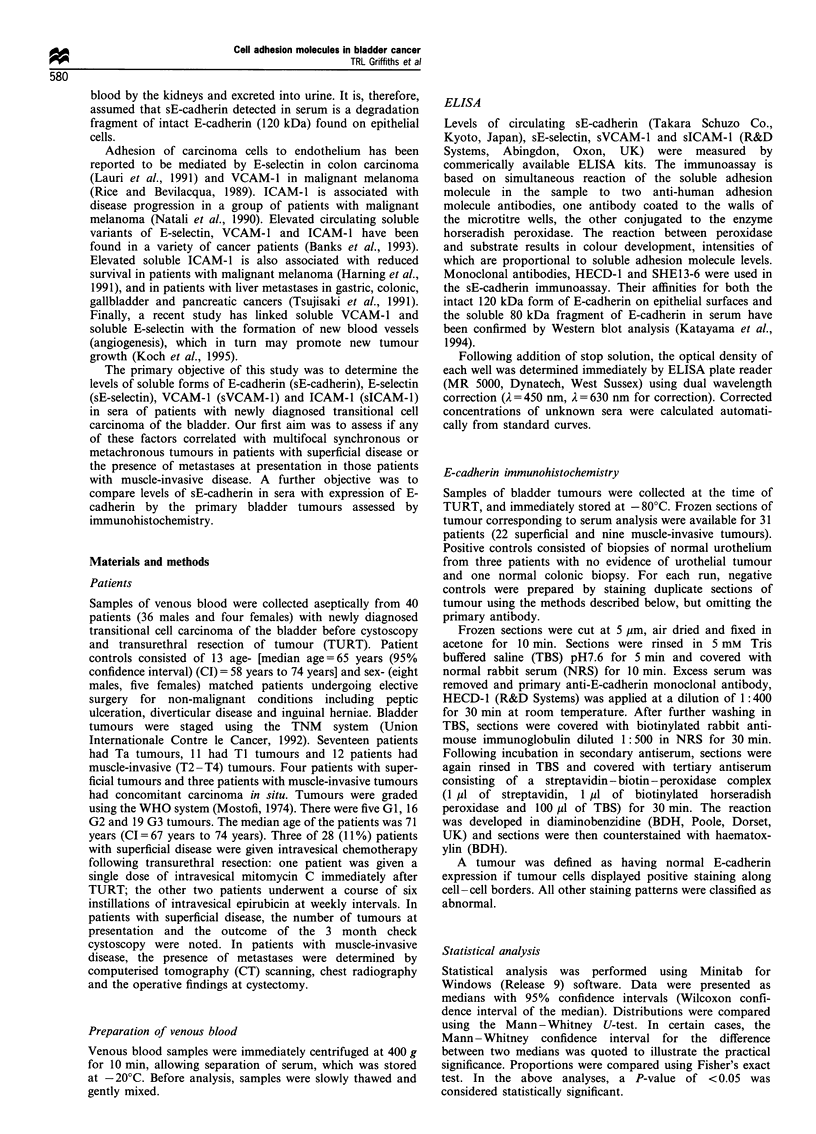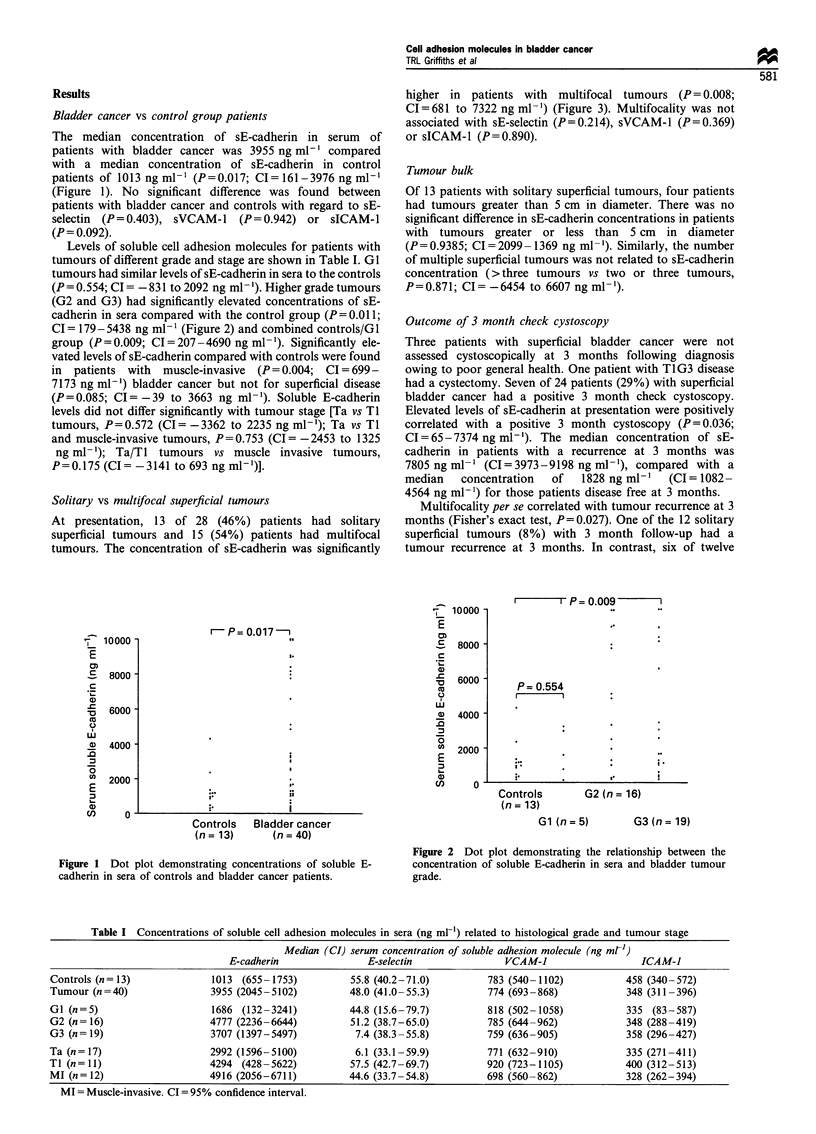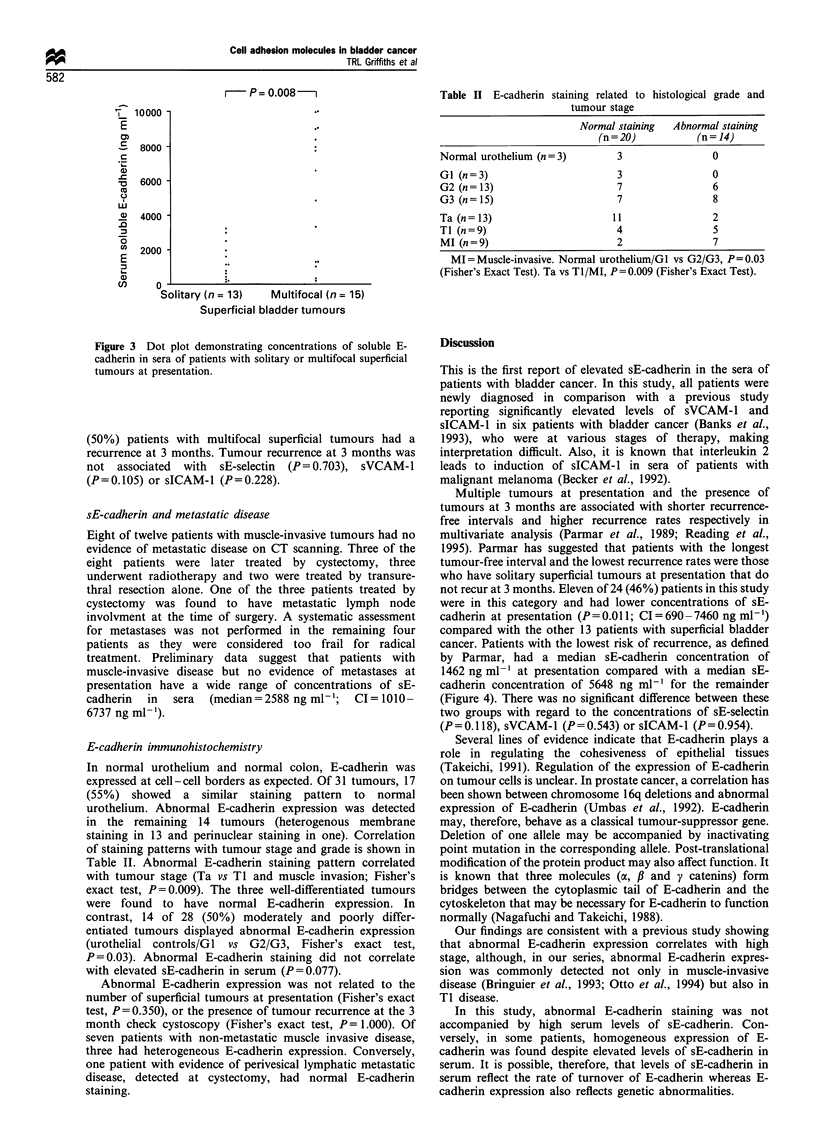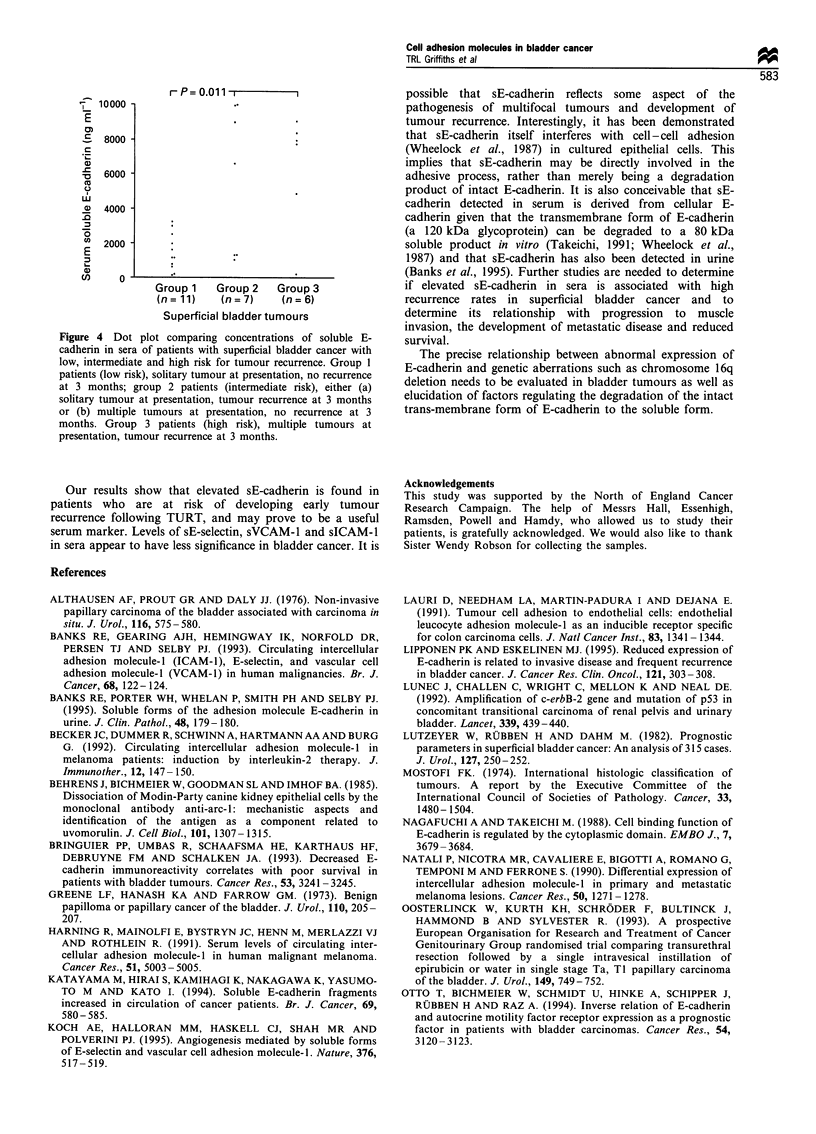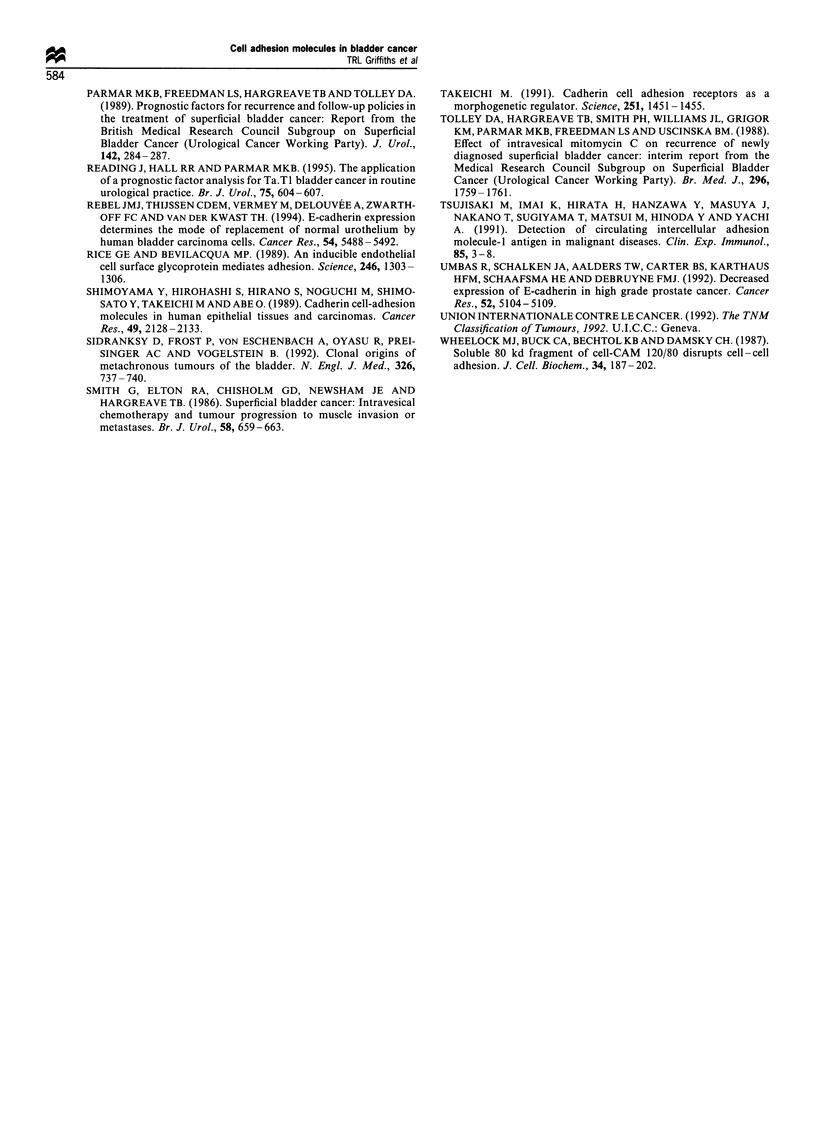# Cell adhesion molecules in bladder cancer: soluble serum E-cadherin correlates with predictors of recurrence.

**DOI:** 10.1038/bjc.1996.404

**Published:** 1996-08

**Authors:** T. R. Griffiths, I. Brotherick, R. I. Bishop, M. D. White, D. M. McKenna, C. H. Horne, B. K. Shenton, D. E. Neal, J. K. Mellon

**Affiliations:** Department of Urology, Freeman Hospital, Newcastle upon Tyne, UK.

## Abstract

Sera from 40 patients with newly diagnosed bladder cancer (28 superficial tumours (pTa and pT1) and 12 muscle-invasive tumours) were assessed by enzyme-linked immunosorbent assay (ELISA) to determine the concentrations of soluble E-cadherin (sE-cadherin), soluble E-selectin (sE-selectin), soluble vascular cell adhesion molecule-1 (sVCAM-1) and soluble intercellular adhesion molecule-1 (sICAM-1). Corresponding frozen sections of primary tumour were analysed for E-cadherin expression using the monoclonal antibody, HECD-1 and standard immunohistochemistry. Patients with bladder cancer had significantly higher concentrations of sE-cadherin compared with a control group (P = 0.017). No difference was found between the two groups with regard to sE-selection (P = 0.403), sVCAM-1 (P = 0.942) and sICAM-1 (P = 0.092). High levels of sE-cadherin were related to poor histological grade (P = 0.009), number of superficial tumours at presentation (P = 0.008) and a positive 3 month check cytoscopy in superficial disease (P = 0.036). Abnormal E-cadherin expression was associated with increasing tumour stage (P = 0.009) and grade (P = 0.03). There was no correlation between high levels of soluble E-cadherin in sera and abnormal E-cadherin expression by the tumour (P = 0.077). Elevated levels of sE-cadherin are found in sera of patients with bladder cancer and correlate with known prognostic factors.


British Journal of Cancer (1996) 74, 579-584
© 1996 Stockton Press All rights reserved 0007-0920/96 $12.00

### Cell adhesion molecules in bladder cancer: soluble serum E-cadherin correlates with predictors of recurrence

TRL Griffiths[1,2], I Brotherick[2], RI Bishop[3], MD White[2], DM McKenna[3], CHW Horne[3], BK Shenton[2], DE Neal[1,2] and JK Mellon[2,4]

[1]*Department of Urology, Freeman Hospital, Freeman Rd, Newcastle upon Tyne NE7 7DN;* [2]*Department of Surgery, The Medical School, Framlington Place, University of Newcastle upon Tyne, Newcastle upon Tyne NE2 4HH;* [3]*School of Pathological Sciences, Division of Pathology, Royal Victoria Infirmary, Queen Victoria Rd, Newcastle upon Tyne NE1 4LP;* [4]*Department of Urology, City Hospitals Sunderland NHS Trust, Kayll Rd, Sunderland SR4 7TP, UK.*

**Summary** Sera from 40 patients with newly diagnosed bladder cancer (28 superficial tumours (pTa and pT1) and 12 muscle-invasive tumours) were assessed by enzyme-linked immunosorbent assay (ELISA) to determine the concentrations of soluble E-cadherin (sE-cadherin), soluble E-selectin (sE-selectin), soluble vascular cell adhesion molecule-1 (sVCAM-1) and soluble intercellular adhesion molecule-1 (sICAM-1). Corresponding frozen sections of primary tumour were analysed for E-cadherin expression using the monoclonal antibody, HECD-1 and standard immunohistochemistry. Patients with bladder cancer had significantly higher concentrations of sE-cadherin compared with a control group ($P=0.017$). No difference was found between the two groups with regard to sE-selectin ($P=0.403$), sVCAM-1 ($P=0.942$) and sICAM-1 ($P=0.092$). High levels of sE-cadherin were related to poor histological grade ($P=0.009$), number of superficial tumours at presentation ($P=0.008$) and a positive 3 month check cytoscopy in superficial disease ($P=0.036$). Abnormal E-cadherin expression was associated with increasing tumour stage ($P=0.009$) and grade ($P=0.03$). There was no correlation between high levels of soluble E-cadherin in sera and abnormal E-cadherin expression by the tumour ($P=0.077$). Elevated levels of sE-cadherin are found in sera of patients with bladder cancer and correlate with known prognostic factors.

**Keywords:** cell adhesion molecule; bladder neoplasm; enzyme-linked immunosorbent assay; immunohistochemistry

Of newly diagnosed superficial bladder tumours, approximately 30% are multifocal at presentation (Lutzeyer *et al.*, 1982), 60-70% will recur (Greene *et al.*, 1973) and 10-20% will undergo stage progression (Lutzeyer *et al.*, 1982). Intense debate surrounds the exact aetiology of synchronous and metachronous bladder cancer. Mucosal abnormalities, namely dysplasia and carcinoma *in situ*, adjacent and distal to the primary tumour, are associated with a poor prognosis, in terms of tumour recurrence and progression in superficial disease (Althausen *et al.*, 1976; Smith *et al.*, 1986). In contrast, recent reports, based on molecular biological studies (Sidransky *et al.*, 1992; Lunec *et al.*, 1992) have added a new dimension suggesting a common clonal origin for concomitant urothelial tumours, at least in some cases. Lateral intraepithelial spread of transformed cells from the origin of a bladder carcinoma or dispersal of tumour cells are possible mechanisms underlying multifocal disease. In support of these theories, a single instillation of intravesical mitomycin C (Tolley *et al.*, 1988) or epirubicin (Oosterlinck *et al.*, 1993) immediately following transurethral resection has been reported to increase time to first recurrence.

Decreased intercellular adhesiveness favours detachment of tumour cells (Takeichi, 1991) and this may play a role in multifocality, the development of recurrent superficial tumours and progression to metastatic disease. At least four families of cell adhesion molecules are thought to be involved in cell-cell adhesion (cadherins, selectins, immunoglobulins and integrins). The most widely studied have been E-cadherin, a cell-surface glycoprotein restricted to epithelial tissue (Behrens *et al.*, 1985; Shimoyama *et al.*, 1989), involved in calcium-dependent homotypic cell-cell adhesion, and vascular cell adhesion molecules [E-selectin, vascular cell adhesion molecule 1; (VCAM-1) and intercellular adhesion molecule 1 (ICAM-1)].

In normal urothelial cells, E-cadherin is homogenously expressed at all cell-cell borders (Bringuier *et al.*, 1993) but not by the cell border in contact with the basement membrane. Loss of expression of E-cadherin on cell membranes has been reported in patients with high-grade, muscle-invasive bladder tumours (Bringuier *et al.*, 1993; Otto *et al.*, 1994; Lipponen and Eskelinen, 1995) and high-grade prostate cancers (Umbas *et al.*, 1992), in which abnormal immunohistochemical patterns including cytoplasmic staining, heterogenous cell membrane expression and negative staining have been reported. Abnormal expression of E-cadherin is associated with reduced recurrence-free survival (Lipponen *et al.*, 1995), along with higher progression rates in patients with superficial bladder tumours (Otto *et al.*, 1994) and also reduced survival in patients with muscle-invasive tumours (Bringuier *et al.*, 1993). It has been suggested that reduced E-cadherin expression, associated with an increase in autocrine motility factor receptor (gp78) expression identifies a group of patients with superficial bladder cancer at high risk of stage progression and earlier cancer-related death (Otto *et al.*, 1994). Using bladder carcinoma cell lines and primary murine urothelium as a model for tumour recurrence, E-cadherin appears to be an important determinant of the pattern of intraepithelial expansion and the ability of tumour cells to colonise primary urothelium (Rebel *et al.*, 1994).

Soluble forms of E-cadherin (sE-cadherin) have been detected in the urine of healthy individuals and patients with bladder cancer (Banks *et al.*, 1995). Soluble E-cadherin fragments have also been found in the sera of patients with gastric and hepatocellular carcinomas (Katayama *et al.*, 1994). The molecular weight of the main form of sE-cadherin detected in urine, 80 kDa, was consistent with that found in serum in the latter study. Furthermore, an 80 kDa fragment is unlikely to represent a product filtered from the

Correspondence: JK Mellon, City Hospitals Sunderland NHS Trust, Kayll Road, Sunderland SR4 7TP, UK
Received 10 November 1995; revised 26 February 1996; accepted 7 March 1996

blood by the kidneys and excreted into urine. It is, therefore, assumed that sE-cadherin detected in serum is a degradation fragment of intact E-cadherin (120 kDa) found on epithelial cells.

Adhesion of carcinoma cells to endothelium has been reported to be mediated by E-selectin in colon carcinoma (Lauri *et al.*, 1991) and VCAM-1 in malignant melanoma (Rice and Bevilacqua, 1989). ICAM-1 is associated with disease progression in a group of patients with malignant melanoma (Natali *et al.*, 1990). Elevated circulating soluble variants of E-selectin, VCAM-1 and ICAM-1 have been found in a variety of cancer patients (Banks *et al.*, 1993). Elevated soluble ICAM-1 is also associated with reduced survival in patients with malignant melanoma (Harning *et al.*, 1991), and in patients with liver metastases in gastric, colonic, gallbladder and pancreatic cancers (Tsujisaki *et al.*, 1991). Finally, a recent study has linked soluble VCAM-1 and soluble E-selectin with the formation of new blood vessels (angiogenesis), which in turn may promote new tumour growth (Koch *et al.*, 1995).

The primary objective of this study was to determine the levels of soluble forms of E-cadherin (sE-cadherin), E-selectin (sE-selectin), VCAM-1 (sVCAM-1) and ICAM-1 (sICAM-1) in sera of patients with newly diagnosed transitional cell carcinoma of the bladder. Our first aim was to assess if any of these factors correlated with multifocal synchronous or metachronous tumours in patients with superficial disease or the presence of metastases at presentation in those patients with muscle-invasive disease. A further objective was to compare levels of sE-cadherin in sera with expression of E-cadherin by the primary bladder tumours assessed by immunohistochemistry.

### Materials and methods

### *Patients*

Samples of venous blood were collected aseptically from 40 patients (36 males and four females) with newly diagnosed transitional cell carcinoma of the bladder before cystoscopy and transurethral resection of tumour (TURT). Patient controls consisted of 13 age- [median age = 65 years (95% confidence interval) (CI) = 58 years to 74 years] and sex- (eight males, five females) matched patients undergoing elective surgery for non-malignant conditions including peptic ulceration, diverticular disease and inguinal herniae. Bladder tumours were staged using the TNM system (Union Internationale Contre le Cancer, 1992). Seventeen patients had Ta tumours, 11 had T1 tumours and 12 patients had muscle-invasive (T2–T4) tumours. Four patients with superficial tumours and three patients with muscle-invasive tumours had concomitant carcinoma *in situ*. Tumours were graded using the WHO system (Mostofi, 1974). There were five G1, 16 G2 and 19 G3 tumours. The median age of the patients was 71 years (CI = 67 years to 74 years). Three of 28 (11%) patients with superficial disease were given intravesical chemotherapy following transurethral resection: one patient was given a single dose of intravesical mitomycin C immediately after TURT; the other two patients underwent a course of six instillations of intravesical epirubicin at weekly intervals. In patients with superficial disease, the number of tumours at presentation and the outcome of the 3 month check cystoscopy were noted. In patients with muscle-invasive disease, the presence of metastases were determined by computerised tomography (CT) scanning, chest radiography and the operative findings at cystectomy.

### *Preparation of venous blood*

Venous blood samples were immediately centrifuged at 400 *g* for 10 min, allowing separation of serum, which was stored at −20°C. Before analysis, samples were slowly thawed and gently mixed.

### *ELISA*

Levels of circulating sE-cadherin (Takara Schuzo Co., Kyoto, Japan), sE-selectin, sVCAM-1 and sICAM-1 (R&D Systems, Abingdon, Oxon, UK) were measured by commerically available ELISA kits. The immunoassay is based on simultaneous reaction of the soluble adhesion molecule in the sample to two anti-human adhesion molecule antibodies, one antibody coated to the walls of the microtitre wells, the other conjugated to the enzyme horseradish peroxidase. The reaction between peroxidase and substrate results in colour development, intensities of which are proportional to soluble adhesion molecule levels. Monoclonal antibodies, HECD-1 and SHE13-6 were used in the sE-cadherin immunoassay. Their affinities for both the intact 120 kDa form of E-cadherin on epithelial surfaces and the soluble 80 kDa fragment of E-cadherin in serum have been confirmed by Western blot analysis (Katayama *et al.*, 1994).

Following addition of stop solution, the optical density of each well was determined immediately by ELISA plate reader (MR 5000, Dynatech, West Sussex) using dual wavelength correction ($\lambda = 450$ nm, $\lambda = 630$ nm for correction). Corrected concentrations of unknown sera were calculated automatically from standard curves.

### *E-cadherin immunohistochemistry*

Samples of bladder tumours were collected at the time of TURT, and immediately stored at −80°C. Frozen sections of tumour corresponding to serum analysis were available for 31 patients (22 superficial and nine muscle-invasive tumours). Positive controls consisted of biopsies of normal urothelium from three patients with no evidence of urothelial tumour and one normal colonic biopsy. For each run, negative controls were prepared by staining duplicate sections of tumour using the methods described below, but omitting the primary antibody.

Frozen sections were cut at 5 $\mu$m, air dried and fixed in acetone for 10 min. Sections were rinsed in 5 mM Tris buffered saline (TBS) pH7.6 for 5 min and covered with normal rabbit serum (NRS) for 10 min. Excess serum was removed and primary anti-E-cadherin monoclonal antibody, HECD-1 (R&D Systems) was applied at a dilution of 1 : 400 for 30 min at room temperature. After further washing in TBS, sections were covered with biotinylated rabbit anti-mouse immunoglobulin diluted 1 : 500 in NRS for 30 min. Following incubation in secondary antiserum, sections were again rinsed in TBS and covered with tertiary antiserum consisting of a streptavidin–biotin–peroxidase complex (1 $\mu$l of streptavidin, 1 $\mu$l of biotinylated horseradish peroxidase and 100 $\mu$l of TBS) for 30 min. The reaction was developed in diaminobenzidine (BDH, Poole, Dorset, UK) and sections were then counterstained with haematoxylin (BDH).

A tumour was defined as having normal E-cadherin expression if tumour cells displayed positive staining along cell–cell borders. All other staining patterns were classified as abnormal.

### *Statistical analysis*

Statistical analysis was performed using Minitab for Windows (Release 9) software. Data were presented as medians with 95% confidence intervals (Wilcoxon confidence interval of the median). Distributions were compared using the Mann–Whitney *U*-test. In certain cases, the Mann–Whitney confidence interval for the difference between two medians was quoted to illustrate the practical significance. Proportions were compared using Fisher's exact test. In the above analyses, a *P*-value of $<0.05$ was considered statistically significant.

### Results

*Bladder cancer* vs *control group patients*

The median concentration of sE-cadherin in serum of patients with bladder cancer was 3955 ng $ml^{-1}$ compared with a median concentration of sE-cadherin in control patients of 1013 ng $ml^{-1}$ ($P=0.017$; CI = 161–3976 ng $ml^{-1}$ (Figure 1). No significant difference was found between patients with bladder cancer and controls with regard to sE-selectin ($P=0.403$), sVCAM-1 ($P=0.942$) or sICAM-1 ($P=0.092$).

Levels of soluble cell adhesion molecules for patients with tumours of different grade and stage are shown in Table I. G1 tumours had similar levels of sE-cadherin in sera to the controls ($P=0.554$; CI = −831 to 2092 ng $ml^{-1}$). Higher grade tumours (G2 and G3) had significantly elevated concentrations of sE-cadherin in sera compared with the control group ($P=0.011$; CI = 179–5438 ng $ml^{-1}$ (Figure 2) and combined controls/G1 group ($P=0.009$; CI = 207–4690 ng $ml^{-1}$). Significantly elevated levels of sE-cadherin compared with controls were found in patients with muscle-invasive ($P=0.004$; CI = 699–7173 ng $ml^{-1}$) bladder cancer but not for superficial disease ($P=0.085$; CI = −39 to 3663 ng $ml^{-1}$). Soluble E-cadherin levels did not differ significantly with tumour stage [Ta *vs* T1 tumours, $P=0.572$ (CI = −3362 to 2235 ng $ml^{-1}$); Ta *vs* T1 and muscle-invasive tumours, $P=0.753$ (CI = −2453 to 1325 ng $ml^{-1}$); Ta/T1 tumours *vs* muscle invasive tumours, $P=0.175$ (CI = −3141 to 693 ng $ml^{-1}$)].

*Solitary* vs *multifocal superficial tumours*

At presentation, 13 of 28 (46%) patients had solitary superficial tumours and 15 (54%) patients had multifocal tumours. The concentration of sE-cadherin was significantly higher in patients with multifocal tumours ($P=0.008$; CI = 681 to 7322 ng $ml^{-1}$) (Figure 3). Multifocality was not associated with sE-selectin ($P=0.214$), sVCAM-1 ($P=0.369$) or sICAM-1 ($P=0.890$).

*Tumour bulk*

Of 13 patients with solitary superficial tumours, four patients had tumours greater than 5 cm in diameter. There was no significant difference in sE-cadherin concentrations in patients with tumours greater or less than 5 cm in diameter ($P=0.9385$; CI = 2099–1369 ng $ml^{-1}$). Similarly, the number of multiple superficial tumours was not related to sE-cadherin concentration (> three tumours *vs* two or three tumours, $P=0.871$; CI = −6454 to 6607 ng $ml^{-1}$).

*Outcome of 3 month check cystoscopy*

Three patients with superficial bladder cancer were not assessed cystoscopically at 3 months following diagnosis owing to poor general health. One patient with T1G3 disease had a cystectomy. Seven of 24 patients (29%) with superficial bladder cancer had a positive 3 month check cystoscopy. Elevated levels of sE-cadherin at presentation were positively correlated with a positive 3 month cystoscopy ($P=0.036$; CI = 65–7374 ng $ml^{-1}$). The median concentration of sE-cadherin in patients with a recurrence at 3 months was 7805 ng $ml^{-1}$ (CI = 3973–9198 ng $ml^{-1}$), compared with a median concentration of 1828 ng $ml^{-1}$ (CI = 1082–4564 ng $ml^{-1}$) for those patients disease free at 3 months.

Multifocality *per se* correlated with tumour recurrence at 3 months (Fisher's exact test, $P=0.027$). One of the 12 solitary superficial tumours (8%) with 3 month follow-up had a tumour recurrence at 3 months. In contrast, six of twelve

**Figure 1** Dot plot demonstrating concentrations of soluble E-cadherin in sera of controls and bladder cancer patients.

**Figure 2** Dot plot demonstrating the relationship between the concentration of soluble E-cadherin in sera and bladder tumour grade.

**Table I** Concentrations of soluble cell adhesion molecules in sera (ng $ml^{-1}$) related to histological grade and tumour stage

| | *Median (CI) serum concentration of soluble adhesion molecule (ng ml⁻¹)* | | | |
|---|---|---|---|---|
| | *E-cadherin* | *E-selectin* | *VCAM-1* | *ICAM-1* |
| Controls ($n=13$) | 1013 (655–1753) | 55.8 (40.2–71.0) | 783 (540–1102) | 458 (340–572) |
| Tumour ($n=40$) | 3955 (2045–5102) | 48.0 (41.0–55.3) | 774 (693–868) | 348 (311–396) |
| G1 ($n=5$) | 1686 (132–3241) | 44.8 (15.6–79.7) | 818 (502–1058) | 335 (83–587) |
| G2 ($n=16$) | 4777 (2236–6644) | 51.2 (38.7–65.0) | 785 (644–962) | 348 (288–419) |
| G3 ($n=19$) | 3707 (1397–5497) | 7.4 (38.3–55.8) | 759 (636–905) | 358 (296–427) |
| Ta ($n=17$) | 2992 (1596–5100) | 6.1 (33.1–59.9) | 771 (632–910) | 335 (271–411) |
| T1 ($n=11$) | 4294 (428–5622) | 57.5 (42.7–69.7) | 920 (723–1105) | 400 (312–513) |
| MI ($n=12$) | 4916 (2056–6711) | 44.6 (33.7–54.8) | 698 (560–862) | 328 (262–394) |

MI = Muscle-invasive. CI = 95% confidence interval.

**Figure 3** Dot plot demonstrating concentrations of soluble E-cadherin in sera of patients with solitary or multifocal superficial tumours at presentation.

(50%) patients with multifocal superficial tumours had a recurrence at 3 months. Tumour recurrence at 3 months was not associated with sE-selectin ($P=0.703$), sVCAM-1 ($P=0.105$) or sICAM-1 ($P=0.228$).

### *sE-cadherin and metastatic disease*

Eight of twelve patients with muscle-invasive tumours had no evidence of metastatic disease on CT scanning. Three of the eight patients were later treated by cystectomy, three underwent radiotherapy and two were treated by transurethral resection alone. One of the three patients treated by cystectomy was found to have metastatic lymph node involvment at the time of surgery. A systematic assessment for metastases was not performed in the remaining four patients as they were considered too frail for radical treatment. Preliminary data suggest that patients with muscle-invasive disease but no evidence of metastases at presentation have a wide range of concentrations of sE-cadherin in sera (median = 2588 ng ml$^{-1}$; CI = 1010–6737 ng ml$^{-1}$).

### *E-cadherin immunohistochemistry*

In normal urothelium and normal colon, E-cadherin was expressed at cell–cell borders as expected. Of 31 tumours, 17 (55%) showed a similar staining pattern to normal urothelium. Abnormal E-cadherin expression was detected in the remaining 14 tumours (heterogenous membrane staining in 13 and perinuclear staining in one). Correlation of staining patterns with tumour stage and grade is shown in Table II. Abnormal E-cadherin staining pattern correlated with tumour stage (Ta *vs* T1 and muscle invasion; Fisher's exact test, $P=0.009$). The three well-differentiated tumours were found to have normal E-cadherin expression. In contrast, 14 of 28 (50%) moderately and poorly differentiated tumours displayed abnormal E-cadherin expression (urothelial controls/G1 *vs* G2/G3, Fisher's exact test, $P=0.03$). Abnormal E-cadherin staining did not correlate with elevated sE-cadherin in serum ($P=0.077$).

Abnormal E-cadherin expression was not related to the number of superficial tumours at presentation (Fisher's exact test, $P=0.350$), or the presence of tumour recurrence at the 3 month check cystoscopy (Fisher's exact test, $P=1.000$). Of seven patients with non-metastatic muscle invasive disease, three had heterogeneous E-cadherin expression. Conversely, one patient with evidence of perivesical lymphatic metastatic disease, detected at cystectomy, had normal E-cadherin staining.

**Table II** E-cadherin staining related to histological grade and tumour stage

| | *Normal staining* (n = *20*) | *Abnormal staining* (n = *14*) |
|---|---|---|
| Normal urothelium ($n=3$) | 3 | 0 |
| G1 ($n=3$) | 3 | 0 |
| G2 ($n=13$) | 7 | 6 |
| G3 ($n=15$) | 7 | 8 |
| Ta ($n=13$) | 11 | 2 |
| T1 ($n=9$) | 4 | 5 |
| MI ($n=9$) | 2 | 7 |

MI = Muscle-invasive. Normal urothelium/G1 vs G2/G3, $P=0.03$ (Fisher's Exact Test). Ta vs T1/MI, $P=0.009$ (Fisher's Exact Test).

### Discussion

This is the first report of elevated sE-cadherin in the sera of patients with bladder cancer. In this study, all patients were newly diagnosed in comparison with a previous study reporting significantly elevated levels of sVCAM-1 and sICAM-1 in six patients with bladder cancer (Banks *et al.*, 1993), who were at various stages of therapy, making interpretation difficult. Also, it is known that interleukin 2 leads to induction of sICAM-1 in sera of patients with malignant melanoma (Becker *et al.*, 1992).

Multiple tumours at presentation and the presence of tumours at 3 months are associated with shorter recurrence-free intervals and higher recurrence rates respectively in multivariate analysis (Parmar *et al.*, 1989; Reading *et al.*, 1995). Parmar has suggested that patients with the longest tumour-free interval and the lowest recurrence rates were those who have solitary superficial tumours at presentation that do not recur at 3 months. Eleven of 24 (46%) patients in this study were in this category and had lower concentrations of sE-cadherin at presentation ($P=0.011$; CI = 690–7460 ng ml$^{-1}$) compared with the other 13 patients with superficial bladder cancer. Patients with the lowest risk of recurrence, as defined by Parmar, had a median sE-cadherin concentration of 1462 ng ml$^{-1}$ at presentation compared with a median sE-cadherin concentration of 5648 ng ml$^{-1}$ for the remainder (Figure 4). There was no significant difference between these two groups with regard to the concentrations of sE-selectin ($P=0.118$), sVCAM-1 ($P=0.543$) or sICAM-1 ($P=0.954$).

Several lines of evidence indicate that E-cadherin plays a role in regulating the cohesiveness of epithelial tissues (Takeichi, 1991). Regulation of the expression of E-cadherin on tumour cells is unclear. In prostate cancer, a correlation has been shown between chromosome 16q deletions and abnormal expression of E-cadherin (Umbas *et al.*, 1992). E-cadherin may, therefore, behave as a classical tumour-suppressor gene. Deletion of one allele may be accompanied by inactivating point mutation in the corresponding allele. Post-translational modification of the protein product may also affect function. It is known that three molecules ($\alpha$, $\beta$ and $\gamma$ catenins) form bridges between the cytoplasmic tail of E-cadherin and the cytoskeleton that may be necessary for E-cadherin to function normally (Nagafuchi and Takeichi, 1988).

Our findings are consistent with a previous study showing that abnormal E-cadherin expression correlates with high stage, although, in our series, abnormal E-cadherin expression was commonly detected not only in muscle-invasive disease (Bringuier *et al.*, 1993; Otto *et al.*, 1994) but also in T1 disease.

In this study, abnormal E-cadherin staining was not accompanied by high serum levels of sE-cadherin. Conversely, in some patients, homogeneous expression of E-cadherin was found despite elevated levels of sE-cadherin in serum. It is possible, therefore, that levels of sE-cadherin in serum reflect the rate of turnover of E-cadherin whereas E-cadherin expression also reflects genetic abnormalities.

**Figure 4** Dot plot comparing concentrations of soluble E-cadherin in sera of patients with superficial bladder cancer with low, intermediate and high risk for tumour recurrence. Group 1 patients (low risk), solitary tumour at presentation, no recurrence at 3 months; group 2 patients (intermediate risk), either (a) solitary tumour at presentation, tumour recurrence at 3 months or (b) multiple tumours at presentation, no recurrence at 3 months. Group 3 patients (high risk), multiple tumours at presentation, tumour recurrence at 3 months.

Our results show that elevated sE-cadherin is found in patients who are at risk of developing early tumour recurrence following TURT, and may prove to be a useful serum marker. Levels of sE-selectin, sVCAM-1 and sICAM-1 in sera appear to have less significance in bladder cancer. It is possible that sE-cadherin reflects some aspect of the pathogenesis of multifocal tumours and development of tumour recurrence. Interestingly, it has been demonstrated that sE-cadherin itself interferes with cell–cell adhesion (Wheelock *et al.*, 1987) in cultured epithelial cells. This implies that sE-cadherin may be directly involved in the adhesive process, rather than merely being a degradation product of intact E-cadherin. It is also conceivable that sE-cadherin detected in serum is derived from cellular E-cadherin given that the transmembrane form of E-cadherin (a 120 kDa glycoprotein) can be degraded to a 80 kDa soluble product *in vitro* (Takeichi, 1991; Wheelock *et al.*, 1987) and that sE-cadherin has also been detected in urine (Banks *et al.*, 1995). Further studies are needed to determine if elevated sE-cadherin in sera is associated with high recurrence rates in superficial bladder cancer and to determine its relationship with progression to muscle invasion, the development of metastatic disease and reduced survival.

The precise relationship between abnormal expression of E-cadherin and genetic aberrations such as chromosome 16q deletion needs to be evaluated in bladder tumours as well as elucidation of factors regulating the degradation of the intact trans-membrane form of E-cadherin to the soluble form.

**Acknowledgements**
This study was supported by the North of England Cancer Research Campaign. The help of Messrs Hall, Essenhigh, Ramsden, Powell and Hamdy, who allowed us to study their patients, is gratefully acknowledged. We would also like to thank Sister Wendy Robson for collecting the samples.

### References

ALTHAUSEN AF, PROUT GR AND DALY JJ. (1976). Non-invasive papillary carcinoma of the bladder associated with carcinoma *in situ*. *J. Urol.*, **116,** 575–580.

BANKS RE, GEARING AJH, HEMINGWAY IK, NORFOLD DR, PERSEN TJ AND SELBY PJ. (1993). Circulating intercellular adhesion molecule-1 (ICAM-1), E-selectin, and vascular cell adhesion molecule-1 (VCAM-1) in human malignancies. *Br. J. Cancer*, **68,** 122–124.

BANKS RE, PORTER WH, WHELAN P, SMITH PH AND SELBY PJ. (1995). Soluble forms of the adhesion molecule E-cadherin in urine. *J. Clin. Pathol.*, **48,** 179–180.

BECKER JC, DUMMER R, SCHWINN A, HARTMANN AA AND BURG G. (1992). Circulating intercellular adhesion molecule-1 in melanoma patients: induction by interleukin-2 therapy. *J. Immunother.*, **12,** 147–150.

BEHRENS J, BICHMEIER W, GOODMAN SL AND IMHOF BA. (1985). Dissociation of Modin-Party canine kidney epithelial cells by the monoclonal antibody anti-arc-1: mechanistic aspects and identification of the antigen as a component related to uvomorulin. *J. Cell Biol.*, **101,** 1307–1315.

BRINGUIER PP, UMBAS R, SCHAAFSMA HE, KARTHAUS HF, DEBRUYNE FM AND SCHALKEN JA. (1993). Decreased E-cadherin immunoreactivity correlates with poor survival in patients with bladder tumours. *Cancer Res.*, **53,** 3241–3245.

GREENE LF, HANASH KA AND FARROW GM. (1973). Benign papilloma or papillary cancer of the bladder. *J. Urol.*, **110,** 205–207.

HARNING R, MAINOLFI E, BYSTRYN JC, HENN M, MERLAZZI VJ AND ROTHLEIN R. (1991). Serum levels of circulating intercellular adhesion molecule-1 in human malignant melanoma. *Cancer Res.*, **51,** 5003–5005.

KATAYAMA M, HIRAI S, KAMIHAGI K, NAKAGAWA K, YASUMOTO M AND KATO I. (1994). Soluble E-cadherin fragments increased in circulation of cancer patients. *Br. J. Cancer*, **69,** 580–585.

KOCH AE, HALLORAN MM, HASKELL CJ, SHAH MR AND POLVERINI PJ. (1995). Angiogenesis mediated by soluble forms of E-selectin and vascular cell adhesion molecule-1. *Nature*, **376,** 517–519.

LAURI D, NEEDHAM LA, MARTIN-PADURA I AND DEJANA E. (1991). Tumour cell adhesion to endothelial cells: endothelial leucocyte adhesion molecule-1 as an inducible receptor specific for colon carcinoma cells. *J. Natl Cancer Inst.*, **83,** 1341–1344.

LIPPONEN PK AND ESKELINEN MJ. (1995). Reduced expression of E-cadherin is related to invasive disease and frequent recurrence in bladder cancer. *J. Cancer Res. Clin. Oncol.*, **121,** 303–308.

LUNEC J, CHALLEN C, WRIGHT C, MELLON K AND NEAL DE. (1992). Amplification of c-*erb*B-2 gene and mutation of p53 in concomitant transitional carcinoma of renal pelvis and urinary bladder. *Lancet*, **339,** 439–440.

LUTZEYER W, RÜBBEN H AND DAHM M. (1982). Prognostic parameters in superficial bladder cancer: An analysis of 315 cases. *J. Urol.*, **127,** 250–252.

MOSTOFI FK. (1974). International histologic classification of tumours. A report by the Executive Committee of the International Council of Societies of Pathology. *Cancer*, **33,** 1480–1504.

NAGAFUCHI A AND TAKEICHI M. (1988). Cell binding function of E-cadherin is regulated by the cytoplasmic domain. *EMBO J.*, **7,** 3679–3684.

NATALI P, NICOTRA MR, CAVALIERE E, BIGOTTI A, ROMANO G, TEMPONI M AND FERRONE S. (1990). Differential expression of intercellular adhesion molecule-1 in primary and metastatic melanoma lesions. *Cancer Res.*, **50,** 1271–1278.

OOSTERLINCK W, KURTH KH, SCHRÖDER F, BULTINCK J, HAMMOND B AND SYLVESTER R. (1993). A prospective European Organisation for Research and Treatment of Cancer Genitourinary Group randomised trial comparing transurethral resection followed by a single intravesical instillation of epirubicin or water in single stage Ta, T1 papillary carcinoma of the bladder. *J. Urol.*, **149,** 749–752.

OTTO T, BICHMEIER W, SCHMIDT U, HINKE A, SCHIPPER J, RÜBBEN H AND RAZ A. (1994). Inverse relation of E-cadherin and autocrine motility factor receptor expression as a prognostic factor in patients with bladder carcinomas. *Cancer Res.*, **54,** 3120–3123.

PARMAR MKB, FREEDMAN LS, HARGREAVE TB AND TOLLEY DA. (1989). Prognostic factors for recurrence and follow-up policies in the treatment of superficial bladder cancer: Report from the British Medical Research Council Subgroup on Superficial Bladder Cancer (Urological Cancer Working Party). *J. Urol.*, **142,** 284–287.

READING J, HALL RR AND PARMAR MKB. (1995). The application of a prognostic factor analysis for Ta.T1 bladder cancer in routine urological practice. *Br. J. Urol.*, **75,** 604–607.

REBEL JMJ, THIJSSEN CDEM, VERMEY M, DELOUVÉE A, ZWARTHOFF FC AND VAN DER KWAST TH. (1994). E-cadherin expression determines the mode of replacement of normal urothelium by human bladder carcinoma cells. *Cancer Res.*, **54,** 5488–5492.

RICE GE AND BEVILACQUA MP. (1989). An inducible endothelial cell surface glycoprotein mediates adhesion. *Science*, **246,** 1303–1306.

SHIMOYAMA Y, HIROHASHI S, HIRANO S, NOGUCHI M, SHIMOSATO Y, TAKEICHI M AND ABE O. (1989). Cadherin cell-adhesion molecules in human epithelial tissues and carcinomas. *Cancer Res.*, **49,** 2128–2133.

SIDRANKSY D, FROST P, VON ESCHENBACH A, OYASU R, PREISINGER AC AND VOGELSTEIN B. (1992). Clonal origins of metachronous tumours of the bladder. *N. Engl. J. Med.*, **326,** 737–740.

SMITH G, ELTON RA, CHISHOLM GD, NEWSHAM JE AND HARGREAVE TB. (1986). Superficial bladder cancer: Intravesical chemotherapy and tumour progression to muscle invasion or metastases. *Br. J. Urol.*, **58,** 659–663.

TAKEICHI M. (1991). Cadherin cell adhesion receptors as a morphogenetic regulator. *Science*, **251,** 1451–1455.

TOLLEY DA, HARGREAVE TB, SMITH PH, WILLIAMS JL, GRIGOR KM, PARMAR MKB, FREEDMAN LS AND USCINSKA BM. (1988). Effect of intravesical mitomycin C on recurrence of newly diagnosed superficial bladder cancer: interim report from the Medical Research Council Subgroup on Superficial Bladder Cancer (Urological Cancer Working Party). *Br. Med. J.*, **296,** 1759–1761.

TSUJISAKI M, IMAI K, HIRATA H, HANZAWA Y, MASUYA J, NAKANO T, SUGIYAMA T, MATSUI M, HINODA Y AND YACHI A. (1991). Detection of circulating intercellular adhesion molecule-1 antigen in malignant diseases. *Clin. Exp. Immunol.*, **85,** 3–8.

UMBAS R, SCHALKEN JA, AALDERS TW, CARTER BS, KARTHAUS HFM, SCHAAFSMA HE AND DEBRUYNE FMJ. (1992). Decreased expression of E-cadherin in high grade prostate cancer. *Cancer Res.*, **52,** 5104–5109.

UNION INTERNATIONALE CONTRE LE CANCER. (1992). *The TNM Classification of Tumours, 1992.* U.I.C.C.: Geneva.

WHEELOCK MJ, BUCK CA, BECHTOL KB AND DAMSKY CH. (1987). Soluble 80 kd fragment of cell-CAM 120/80 disrupts cell–cell adhesion. *J. Cell. Biochem.*, **34,** 187–202.